# A case of plexiform schwannoma arising from the sciatic, tibial, and peroneal nerves

**DOI:** 10.1016/j.radcr.2023.10.009

**Published:** 2023-11-02

**Authors:** Shuhei Miyamoto, Ryo Takaji, Norimasa Kaneko, Tatsuya Iwasaki, Masanori Kawano, Kazuhiro Tanaka, Tsutomu Daa, Yoshiki Asayama

**Affiliations:** a Departments of Radiology, Oita University Faculty of Medicine, Yufu, Oita 879-5593, Japan; b Departments of Orthopedic Surgery, Oita University Faculty of Medicine, Yufu, Oita 879-5593, Japan; c Departments of Diagnostic Pathology, Oita University Faculty of Medicine, Yufu, Oita 879-5593, Japan

**Keywords:** Magnetic resonance imaging, Peroneal nerve, Plexiform schwannoma, Sciatic nerve, Target sign, Tibial nerve

## Abstract

Plexiform schwannoma is a rare subtype of schwannoma. In this report, we present a case of plexiform schwannoma arising from the sciatic, tibial, and peroneal nerves. A 54-year-old man presented with a painful palpable mass extending from the left posterior thigh to the calf. Magnetic resonance imaging showed multiple bead-like nodular structures along the sciatic, tibial, and peroneal nerve pathway. The nodular lesions showed uniform signal intensity on T1-weighted imaging. On T2-weighted imaging, each nodule showed an eccentric area of relatively low signal intensity surrounded by an area of higher signal intensity and a low-intensity rim. Plexiform schwannoma or neurofibroma was considered as the preoperative diagnosis. Because of the patient's severe symptoms and strong desire for relief, tumor enucleation of the largest painful nodule was performed, and plexiform schwannoma was confirmed pathologically.

## Introduction

Schwannomas are benign tumors derived from Schwann cells of peripheral nerve sheaths. Plexiform schwannoma is a subtype of schwannoma that exhibits a characteristic morphological bead-like or multinodular appearance. This subtype is extremely rare, accounting for less than 5% of all schwannomas [1,2]. Furthermore, plexiform schwannomas arising from major peripheral nerve trunks and deep nerves, such as the sciatic nerve, are even rarer [1]. We herein present a case of multiple plexiform schwannomas arising from the sciatic, tibial, and peroneal nerves with a focus on the magnetic resonance imaging (MRI) features. We also provide a literature-based discussion of plexiform schwannoma.

## Case report

A 54-year-old man presented with a painful palpable mass extending from the left posterior thigh to the calf. He was referred to our orthopedic department for further investigation and treatment. The patient had a history of lumbar intervertebral disc herniation 4 months previously. Blood biochemical tests revealed no significant findings. The patient had no noteworthy family history.

**Fig. 1:**
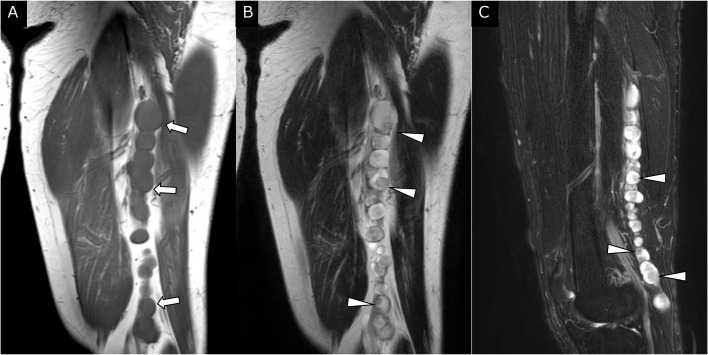
MRI of the left thigh. (A) Coronal T1WI showed a bead-like multinodular structure (arrows) with uniform signal intensity compared with skeletal muscle. (B) Coronal T2WI and (C) sagittal fat-suppressed T2WI showed multiple well-defined bead-like structures with localization corresponding to the sciatic nerve pathway. These nodules showed heterogeneous high signal intensity and had a low-intensity rim. Eccentric intratumoral areas of relatively low signal intensity (arrowheads) were also observed.

**Fig. 2:**
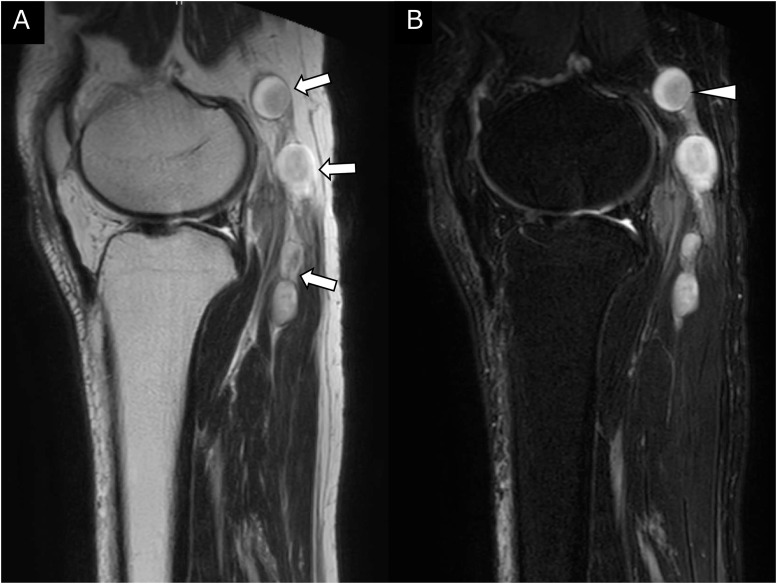
MRI of the left popliteal fossa. (A, B) Sagittal T2WI and fat-suppressed T2WI showed that a multinodular bead-like structure (arrows) was depicted along the tibial and peroneal nerve pathway. The nodules showed heterogenous high signal intensity, and eccentric relatively low-intensity areas (arrowheads) were also observed.

MRI revealed multiple well-defined nodular structures in the left lower limb, extending from the anterior aspect of the quadriceps femoris muscle to the popliteal fossa and proximal lower leg. The nodular structures exhibited a bead-like pattern and measured up to 1.8 × 1.7 cm in size (Fig. 1, Fig. 2). The nodules showed uniform signal intensity comparable to skeletal muscle on T1-weighted imaging (T1WI) (Fig. 1) and heterogeneous high signal intensity on T2-weighted imaging (T2WI) (Figs. 1 and 2) and fat-suppressed T2WI (Figs. 1 and 2). On T2WI, each nodule showed an eccentric area of relatively low signal intensity surrounded by an area of higher signal intensity, and a low-intensity rim was clearly depicted along the periphery of the nodules (Figs. 1 and 2). The location of these multiple nodular lesions corresponded to the pathway of the sciatic, tibial, and peroneal nerves. Plexiform schwannoma or plexiform neurofibroma was considered as the preoperative imaging diagnosis.

Because of the patient's severe pain corresponding to a portion of the tumor in the left calf and his strong desire for relief, the largest tumor in the calf was surgically excised. Intraoperatively, the nerve fibers were observed to partially surround the tumor, and tumor enucleation was performed to preserve the nerve function.

**Fig. 3:**
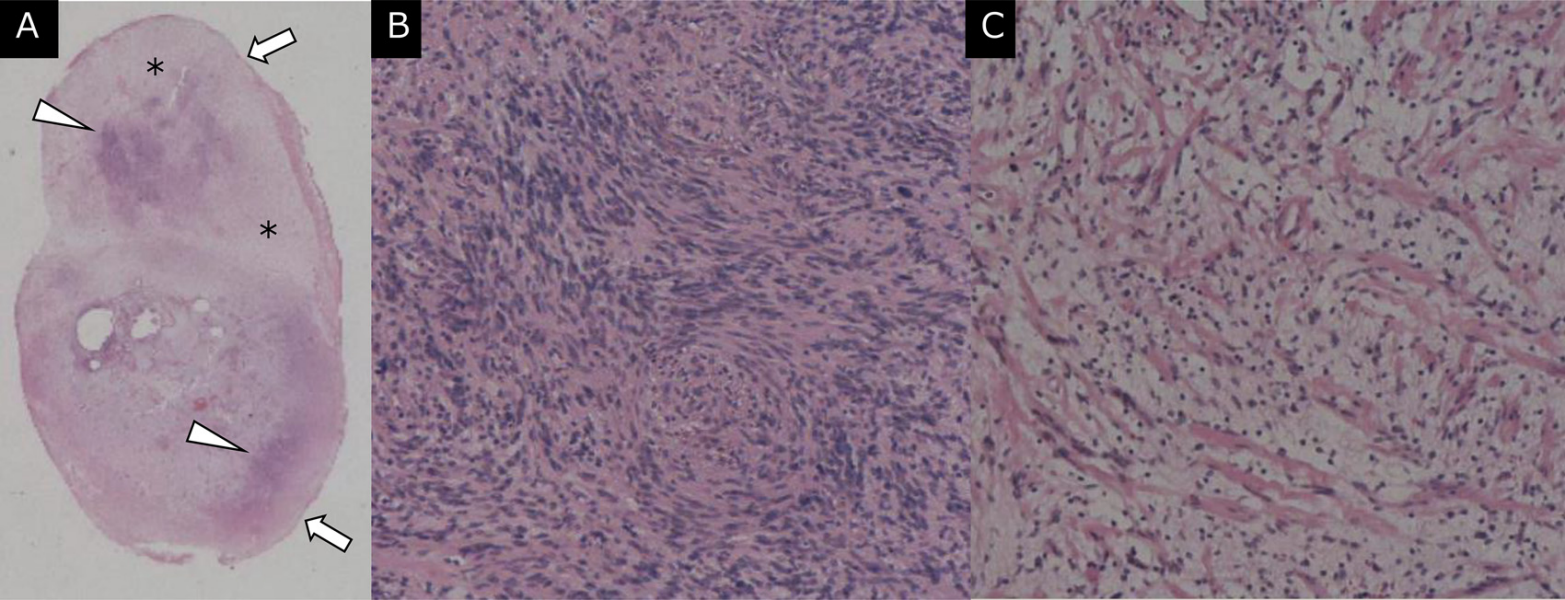
Histological section (hematoxylin and eosin stain) of the enucleated lower limb tumor. (A) Loupe image showed dense (arrowheads) and sparse (*) compositions within the tumor. A fibrous capsule (arrows) was present at the periphery of the tumor. Microscopic examination revealed (B) a dense cellular component containing spindle cell proliferation without an increase in mitotic figures (Antoni A area) and (C) a sparse cellular component consisting of spindle cells with loose myxoid matrix (Antoni B area). Histological images showed typical features of schwannoma.

Gross examination revealed a smooth-surfaced tumor with a yellowish-white appearance. Microscopically, the tumor had a fibrous capsule and was composed of densely clustered spindle-shaped cells arranged in a palisading pattern (Antoni A areas) (Fig. 3), and areas with low cell density were observed (Antoni B areas) (Fig. 3). Based on the radiological and histopathological findings, the diagnosis of plexiform schwannoma was confirmed. No pathological features of malignancy were observed.

## Discussion

Plexiform schwannoma is a rare subtype of schwannoma that proliferates in a bead-like or plexiform pattern along a nerve and accounts for 2%-5% of all schwannomas [1,2]. It was first reported by Masson et al. [3,4] in 1970 as plexiform schwannoglioma, demonstrating a plexiform pattern macroscopically and histologically composed of Schwann cells. In 1978, Harkin et al. [5] reported 6 cases of benign plexiform schwannoma.

Plexiform schwannoma commonly occurs in individuals aged 20-50 years, and there is no sex predilection [6]. Surgical resection is the most common treatment. Most plexiform schwannomas occur in the superficial parts of the skin or subcutaneous tissue; plexiform schwannomas arising from peripheral nerve trunks or deep nerves are rare [1]. Pathologically, each individual nodule is encapsulated by non-neoplastic connective tissue, and the internal nodules are composed of a dense proliferation of spindle-shaped cells arranged in a palisading pattern known as Antoni A areas as well as areas with lower cell density known as Antoni B areas [6,7]. In the present case, a man in his *50s* presented with multiple nodular lesions in the left lower limb, and tumor enucleation was performed for the largest nodule. Multiple nodular lesions arranged in a bead-like pattern along the pathway of the sciatic, tibial, and peroneal nerves were found on preoperative MRI, and the enucleated tumor showed typical pathological findings of schwannoma consisting of Antoni A and B areas. Although the tumors were located along the peripheral nerve trunk, a diagnosis of plexiform schwannoma was made based on both radiological and pathological findings.

Plexiform neurofibroma is a tumor with a morphology similar to that of plexiform schwannoma [8–10]. Plexiform neurofibroma manifest as malignant peripheral nerve sheath tumors in 5%-20% of cases [8–10], whereas malignant transformation of plexiform schwannoma is extremely rare [6]. Therefore, differentiating between plexiform schwannoma and neurofibroma is clinically important. With respect to patients’ backgrounds, most plexiform schwannomas are not associated with neurofibromatosis (NF) type 1 or 2, whereas plexiform neurofibroma occurs in 30%-50% of patients with NF type 1 [11]. The tumor location is not useful for distinguishing between these tumors because both plexiform schwannoma and neurofibroma commonly occur in the superficial parts of the skin or subcutaneous tissue [1,2,8]. Our patient had no clinical features indicating NF type 1 or 2, and the tumor localization along the peripheral nerve trunk was not typical for either plexiform schwannoma or neurofibroma.

On MRI, plexiform schwannoma and neurofibroma show multiple bead-like nodular lesions. Generally, schwannomas have a low-intensity rim reflecting a fibrous capsule at the periphery on T2WI. By contrast, a low-intensity rim on T2WI is less common in neurofibromas [4,12]. Both tumors can exhibit a “target sign” on T2WI, with peripheral high signal intensity and central low signal intensity [9,12,13]. In our case, T2WI revealed encapsulated bead-like multinodular lesions with a target sign in each nodule. Notably, we observed intratumoral areas of low signal intensity with an eccentric distribution. By contrast, dot-like areas of low signal intensity were observed in the centers of the nodules on T2WI in previously reported cases of plexiform neurofibroma [8,10]. This difference is speculated to reflect the pathological features of the target sign. Schwannoma exhibits a heterogeneous mixture of Antoni A areas with relatively low T2 signal intensity reflecting high cell density and Antoni B areas with high T2 signal intensity reflecting low cell density, whereas neurofibroma shows myxoid matrix with high T2 signal intensity surrounding dense nerve fibers with dot-like central low T2 signal intensity [13]. Thus, MRI might be a crucial imaging modality for differentiation of plexiform schwannoma and neurofibroma by focusing on the distribution of intratumoral low-intensity areas on T2WI.

## Conclusions

We experienced a case of plexiform schwannoma arising from the sciatic, tibial, and peroneal nerves. The occurrence of plexiform schwannoma in a peripheral nerve trunk is rare; however, when a bead-like multinodular tumor is present along a nerve pathway, the possibility of plexiform schwannoma should be considered. An eccentric target-like appearance on T2-weighted MRI might be crucial for distinguishing plexiform schwannoma from plexiform neurofibroma.

## Author contributions

All authors provided final approval of the submitted version.

## Patient consent

Informed consent was obtained from the patient for the publication of this report and any accompanying images.
